# Diaphragm and abdominal organ motion during radiotherapy: a comprehensive multicenter study in 189 children

**DOI:** 10.1186/s13014-023-02307-3

**Published:** 2023-07-13

**Authors:** Karin M. Meijer, Irma W. E. M. van Dijk, Marije Frank, Arnout D. van den Hoek, Brian V. Balgobind, Geert O. Janssens, Markus Wendling, John H. Maduro, Abigail Bryce-Atkinson, Anna Loginova, Arjan Bel

**Affiliations:** 1 Department of Radiation Oncology, Cancer Center Amsterdam, Amsterdam UMC, University of Amsterdam, Meibergdreef 9, Amsterdam, 1105 AZ The Netherlands; 2 Princess Máxima Center for Pediatric Oncology, Utrecht, The Netherlands; 3 Department of Radiation Oncology, University Medical Center Utrecht, Utrecht, The Netherlands; 4 Department of Radiation Oncology, Radboud University Medical Center, Nijmegen, The Netherlands; 5 Department of Radiation Oncology, University of Groningen, University Medical Center Groningen, Groningen, The Netherlands; 6 Division of Cancer Sciences, School of Medical Sciences, Faculty of Biology, Medicine and Health, The University of Manchester, Manchester, UK; 7 Dmitry Rogachev National Research Center of Pediatric Hematology, Oncology and Immunology, Moscow, Russia

**Keywords:** Pediatric cancer, Organ motion, Systematic and random errors, Safety margins

## Abstract

**Background:**

For accurate thoracic and abdominal radiotherapy, inter- and intrafractional geometrical uncertainties need to be considered to enable accurate margin sizes. We aim to quantify interfractional diaphragm and abdominal organ position variations, and intrafractional diaphragm motion in a large multicenter cohort of pediatric cancer patients (< 18 years). We investigated the correlation of interfractional position variations and intrafractional motion with age, and with general anesthesia (GA).

**Methods:**

In 189 children (mean age 8.1; range 0.4–17.9 years) from six institutes, interfractional position variation of both hemidiaphragms, spleen, liver, left and right kidneys was quantified using a two-step registration. CBCTs were registered to the reference CT relative to the bony anatomy, followed by organ registration. We calculated the group mean, systematic and random errors (standard deviations Σ and σ, respectively) in cranial-caudal (CC), left-right and anterior-posterior directions. Intrafractional right hemidiaphragm motion was quantified using CBCTs on which the breathing amplitude, defined as the difference between end-inspiration and end-expiration peaks, was assessed (N = 79). We investigated correlations with age (Spearman’s ρ), and differences in motion between patients treated with and without GA (N = 75; all < 5.5 years).

**Results:**

Interfractional group means were largest in CC direction and varied widely between patients, with largest variations in the right hemidiaphragm (range -13.0–17.5 mm). Interfractional group mean of the left kidney showed a borderline significant correlation with age (p = 0.047; ρ = 0.17). Intrafractional right hemidiaphragm motion in patients ≥ 5.5 years (mean 10.3 mm) was significantly larger compared to patients < 5.5 years treated without GA (mean 8.3 mm) (p = 0.02), with smaller Σ and σ values. We found a significant correlation between breathing amplitude and age (p < 0.001; ρ = 0.43). Interfractional right hemidiaphragm position variations were significantly smaller in patients < 5.5 years treated with GA than without GA (p = 0.004), but intrafractional motion showed no significant difference.

**Conclusion:**

In this large multicenter cohort of children undergoing thoracic and abdominal radiotherapy, we found that interfractional position variation does not depend on age, but the use of GA in patients < 5.5 years showed smaller systematic and random errors. Furthermore, our results showed that breathing amplitude increases with age. Moreover, variations between patients advocate the need for a patient-specific margin approach.

**Supplementary Information:**

The online version contains supplementary material available at 10.1186/s13014-023-02307-3.

## Background

Ongoing developments of multimodality treatment for pediatric cancers have led to an increased childhood cancer survival rate over the past decades [1]. With this progress, the risk of developing treatment-related long-term adverse effects also increases, and it has been shown that radiotherapy is a major determinant [2, 3]. This emphasizes the need for highly accurate treatment planning and dose delivery to target volumes, which are challenged by patient set-up variations, interfractional variations (caused by e.g. anatomical day-to-day variations), intrafractional variation (caused by e.g. breathing or peristaltic motion), and delineation variability. Especially for thoracic and abdominal tumors these uncertainties are considerable. Therefore, the gross and clinical target volumes (GTV and CTV, respectively) are expanded with safety margins, thereby defining the planning target volume (PTV) [4]. Similar margins can be calculated for organs at risk (OAR) to define the planning risk volume (PRV), ensuring that OAR dose is not exceeded [5].

So far, guidelines for child-specific safety margins could not be defined, and there is no clear indication if margin sizes can be reduced for children [6]. A number of studies have quantified diaphragm and abdominal position variations in children using (four-dimensional) computed tomography ((4D)CT) and daily cone-beam CT (CBCT) [7–9], and other studies assessed intrafractional motion using 4DCT [6, 8, 10, 11] and 4D magnetic resonance imaging (MRI) [12]. Studies reported smaller interfractional diaphragm and kidney position variations in children compared to adults, resulting in smaller systematic and random errors [7, 9, 10]. However, no correlations between interfractional organ position variations and patient characteristics (e.g. age and height) [7–9] were found. Also, studies reported smaller intrafractional diaphragm and abdominal organ motion in children aged < 8 years compared to older children [6, 12], or adults [10, 13], suggesting smaller safety margins could be applied for (younger) children compared to adults. Because these studies consisted of relatively small cohorts (range 15–45 patients), quantitative data on pediatric organ motion remains limited. Studies subsequently recommended to include larger patient cohorts to improve the statistical power of the analysis before child-specific safety margins could be defined [6, 7, 10, 12, 13]. To date, such a comprehensive analysis using a large data set has not been performed. Moreover, we recently conducted a systematic review on organ motion, margin sizes and delineation variability, and this demonstrated that inter- and intrafractional organ motion during radiotherapy has been assessed using patient cohorts including 4 to 45 children. However, the variations in patient population and quantification methods hampered direct comparison of the results [14].

Therefore, we aimed to quantify and analyze interfractional position variations of the diaphragm and abdominal organs, and intrafractional diaphragm motion using a large international cohort of pediatric cancer patients. Subsequently, we investigated possible correlations of diaphragm or abdominal organ position variations, and diaphragm motion with patient-specific factors, and with general anesthesia (GA).

## Methods

### Multicenter cohort

**Fig. 1:**
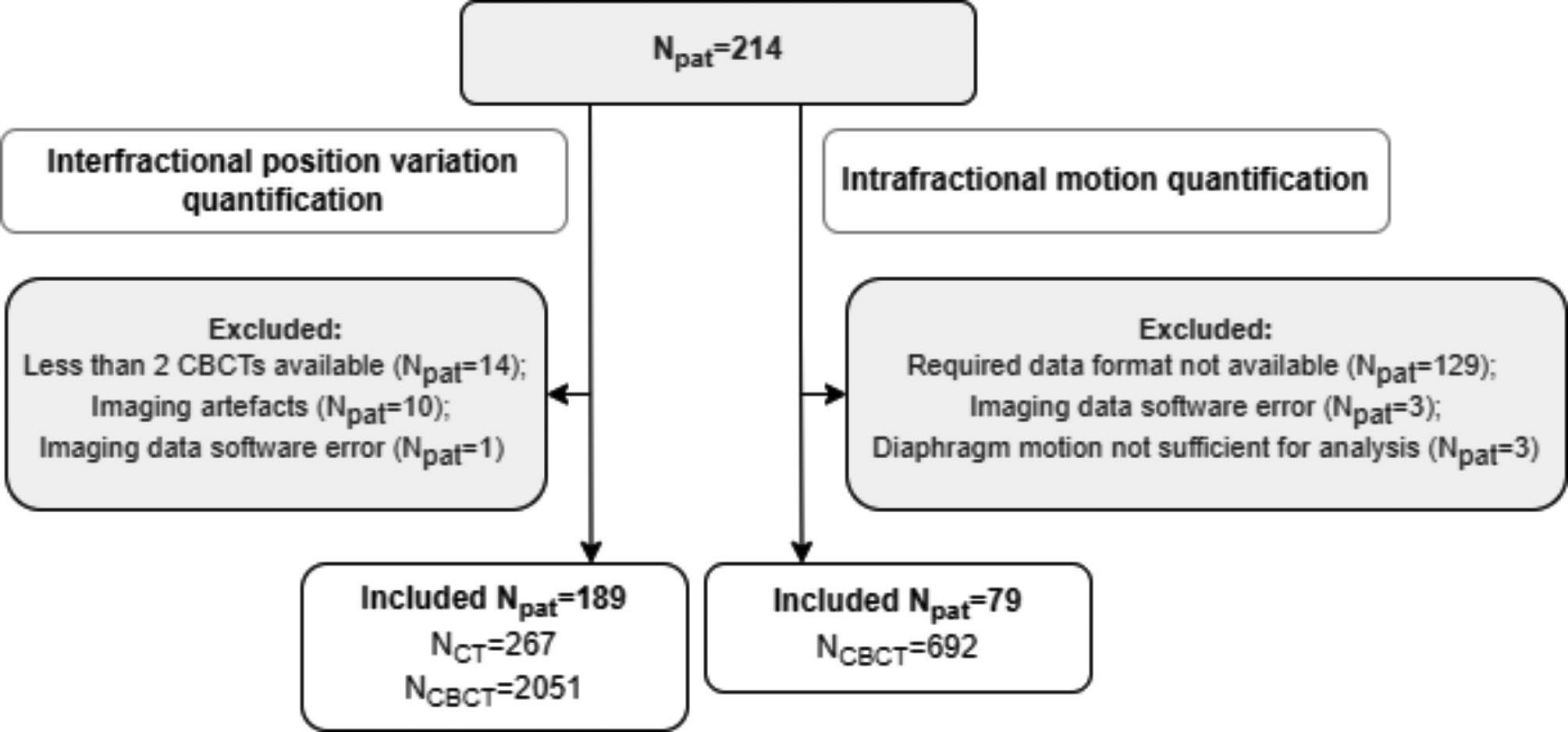
Patient inclusion for interfractional position variation and intrafractional motion analysis

For this retrospective international multicenter study, data of 214 pediatric (< 18 years) patients (N_pat_) whom received radiotherapy in the thoracic and abdominal region between 2010 and 2018, were collected from six institutes. Patients were excluded from interfractional analysis when less than two evaluable CBCTs were available (N_pat_=14), CTs and/or CBCTs showed severe artefacts (N_pat_=10), or an irreversible data error occurred (N_pat_=1), totaling 189 patients included to quantify interfractional position variations (Fig. 1). Patients were included for intrafractional diaphragm motion quantification when the right hemidiaphragm was evaluable on the CBCTs. We quantified intrafractional motion of the right hemidiaphragm only, since cardiac motion may interfere with respiratory motion of the left hemidiaphragm, and consequently may affect the imaging of the breathing cycle and the assessment of the end-inspiration and end-expiration position of the left hemidiaphragm. Patients were excluded from analyses when 2D projection and frame data were not available (N_pat_=129), an imaging data software error occurred (N_pat_=3), or diaphragm motion was too small to track on CBCT imaging and not sufficient for analysis (N_pat_=3). This resulted in 79 patients included for intrafractional motion analyses (Fig. 1). Patient characteristics are described in Table 1. Most patients were treated in supine positioning (185/189; 98.4%), and for a minority of patients a vacuum mattress for immobilization was used (38/189; 20.1%). As the majority of the patients (79.9%) was treated without immobilization, and other factors such as GA were involved, the correlation between immobilization and interfractional position variations and intrafractional motion was not investigated. For 13 patients of whom height and/or weight were not available, the missing data was estimated using national growth diagrams of their corresponding nationality.

**Table 1:** Characteristics of the 189 pediatric patients treated between 2010 and 2018 in the six participating centers

	Interfractional position variation	Intrafractional motion
	N (%)	N (%)
Total	189	79
Male/Female	117/72 (61.9/38.1)	54/25 (68.4/31.6)
Age (years) at first RT fraction		
Mean (SD) [range]	8.1 (4.9) [0.4–17.9]	8.6 (4.4) [1.0–17.8]
0.4 − 5.4	75 (39.7)	23 (29.1)
5.5–17.9	114 (60.3)	56 (70.9)
Height (cm); mean [range]	127.7 [66.0–196.0]	131.5 [74.0–185.0]
Weight (kg); mean [range]	30.9 [6.0–97.5]	32.1 [9.0–97.5]
Type of primary cancer, N (%)^a^		
Neuroblastoma	43 (22.8)	8 (10.1)
Renal tumors	37^b^ (19.6)	6^c^ (7.6)
Medulloblastoma	22 (11.6)	18 (22.8)
Ewing sarcoma	18 (9.5)	6 (7.6)
Leukemia	17 (9.0)	15 (19.0)
Hodgkin lymphoma	16 (8.5)	-
Rhabdomyosarcoma	5 (2.6)	1 (1.3)
Other^d^	26 (13.8)	21 (26.6)
Not reported	5 (2.6)	4 (5.1)
Radiation site		
Craniospinal^e^	45 (23.8)	30 (38.0)
Thoracic/mediastinal^f^	46 (24.3)	14 (17.7)
Abdominal (incl. flank)^f^	90 (47.6)	20 (25.3)
Total body irradiation	20 (10.6)	18 (22.8)
General anesthesia		
N (%)	40 (21.2)	12 (15.2)
Mean age [range] (years)	2.7 [0.5–5.4]	3.3 [1.6–5.1]
N (%)^a^ treated in		
AMC	60^ g^ (31.7)	42^ h^ (53.2)
Christie	11 (5.8)	8 (10.1)
DRC-PHOI	35 (18.5)	29 (36.7)
UMCG	3^ g^ (1.6)	-
UMCN	35^ g^ (18.5)	-
UMCU	45 (23.8)	-

^a^Percentages may not exactly total 100 due to rounding, ^b^N=12 patients with diagnosis Wilms’ tumor, ^c^All N = 6 with Wilms’ tumor, ^d^Other, including: adenocarcinoma (N = 1), atypical teratoid rhabdoid tumor (N = 1), desmoplastic small round cell tumor (N = 3), ependymoma (N = 3), embryonal rhabdomyosarcoma (N = 1), (pineal) germinoma (N = 5), glioma (N = 2), lymphoma (N = 3), myelofibrosis (N = 1), osteosarcoma (N = 1), primitive neuro-ectodermal tumor (N = 1), pleuropulmonary blastoma, (N = 1), undifferentiated sarcoma (N = 3), ^e^N=4 received only spinal irradiation, ^f^N=12 (interfractional) and N = 3 (intrafractional) received radiotherapy at the thoracic *and* abdominal site, ^g^Interfractional position variations of diaphragm and kidneys previously reported for 20/60 (33%) AMC, 3/3 (100%) UMCG and 16/35 (46%) UMCN patients (7), ^h^Patients were previously reported on [11, 13]

RT, Radiotherapy; N, number of patients; AMC, Amsterdam University Medical Center, location AMC; Christie, The Christie NHS Foundation Trust, Manchester; DRC-PHOI, Dmitry Rogachev National Research Center of Pediatric Oncology, Hematology and Immunology Moscow; UMCG, University Medical Center Groningen; UMCN, Radboud University Medical Center Nijmegen; UMCU, University Medical Center Utrecht

### Treatment and imaging data

Pre-treatment CT scans for planning purposes had been acquired according to institution based standard protocols, and were considered as the reference baseline for diaphragm and organ position (refCT). Radiation treatment was delivered with photons using linear accelerators with an integrated CBCT scanner (Synergy, Elekta Oncology Systems, Crawley, UK). Overall, 267 refCTs (N_CT_; range 1–4 per patient, slice thickness 2.0–7.5 mm) of the thorax and/or abdomen were used for interfractional position variation quantification. For different reasons, 60 patients had multiple refCTs; N_pat_=34 had a repeated planning CT during the treatment period, and N_pat_=26 had thoracic as well as abdominal CTs, which were both used for either diaphragm or abdominal organ position variation quantification. For each patient, CBCTs had been acquired; the total number of CBCTs (N_CBCT_) used to quantify interfractional position variation was 2051 (range 2–35 per patient). For intrafractional diaphragm motion quantification, a total of N_CBCT_=692 (range 2–28 per patient) were used, including 331 thoracic and 361 abdominal CBCTs. The CBCT parameters were 100 or 120 kV, 10 to 40 mA with an exposure time of 10 or 40 ms per projection. Gantry rotation varied from 120 to 360 degrees, resulting in a varying number of projection images per CBCT (range 170–760).

### Interfractional position variation

We used a two-step rigid registration to quantify interfractional position variations of the left and right hemidiaphragms, spleen, liver, and the left and right kidneys (when present), using Elekta X-ray Volume Imaging (XVI) software (version 5.0; Elekta Oncology Systems). We will use the term structures to refer to hemidiaphragms and abdominal organs. Not every structure of each of the 189 patients could be evaluated due to artefacts, nephrectomy, or if the structure was not sufficiently visible for registration. First, a region of interest (ROI) was defined including the vertebrae at the level of the diaphragm domes and kidneys. Using the automatic chamfer match algorithm and the defined ROI, the CBCTs were registered to the refCT with respect to the bony anatomy. With this first registration a consistent quantification of organ position with respect to the bony anatomy was feasible. Second, the whole volume of each organ (spleen, liver, left and right kidney) was delineated as a separate ROI, and each organ was automatically registered to the refCT using a grey value algorithm. Whole organ delineation comprises many voxels in the CT volume, therefore, the registration accuracy is higher than the slice thickness and voxel size of the images. Automated registration results were visually inspected and, if necessary, manually corrected. Magnitude and direction of interfractional organ position variations relative to the bony anatomy were assessed by comparing the coordinates of the center of mass (COM) of each organ after registration to the refCT [7, 8]. Abdominal organ position variations were assessed in cranial-caudal (CC), left-right (LR) and anterior-posterior (AP) directions. The + and - signs represent caudal/right/posterior and cranial/left/anterior directions, respectively. The left and right hemidiaphragms were manually registered after the automatic bony anatomy match using the most cranial top of the hemidiaphragm, in CC direction only. This procedure ensured a consistent manual registration. For ten patients the artefacts were too severe, and these patients were excluded from analysis.

### Intrafractional motion

Motion of the right hemidiaphragm in CC direction was extracted using an adapted version of the Amsterdam Shroud (AS) method, which has been described previously [13, 15], and was performed by two experienced observers (KM, SH). In short, a CC gradient filter was applied on a selected ROI, and a 2D AS image was created. On this AS image, the projection images corresponding to end-inspiration and end-expiration positions of the right hemidiaphragm were manually selected. In each of the selected images, we manually assessed the CC position of the top of the hemidiaphragm, resulting in a timeframe describing the CC position of the hemidiaphragm in end-inhale and end-exhale peaks over the course of CBCT acquisition. The amplitude of hemidiaphragm motion was defined as the absolute difference between the averaged end-inspiration peaks and averaged end-expiration peaks.

### Statistical analysis

Per patient, we collected all interfractional position variation results and, regardless of the number of used refCTs, calculated one mean, median and standard deviation (SD) of all organs (when present) in three directions, and of both hemidiaphragms in CC direction only. We reported the interfractional position variation results as the group mean (M), median, range of individual means, group systematic error (Σ; SD of the individual means of all patients) and the group random error (σ; root mean square of the individual SDs of all patients) [4]. Applied equations are described in Additional File 1.1.A.

For intrafractional motion analysis, we calculated the mean breathing amplitude for each fraction. These results were used to calculate per patient the mean amplitude over all fractions, and the variation between fractions (SD over the mean amplitudes of each fraction) were calculated. Then, for all patients, we reported the group mean (M), SD and range of individual breathing amplitudes, and the group random error (Additional File 1.1.B).

For the interfractional position variation and intrafractional motion results of each structure, we used the Shapiro-Wilks test to check for normal data distribution. Since not all data fitted the normal distribution, we used non-parametric tests for all analyses. The one-sample Wilcoxon signed-rank test was used to test if the interfractional group mean position variation of each structure significantly differed from zero, i.e. from the refCT. A dependency between position variation of both hemidiaphragms and abdominal organs can be assumed; therefore a Bonferroni’s corrected *p* value < 0.008 (0.05/6) was considered significant.

For all structures, we used a linear regression analysis (Spearman’s rank correlation coefficient; *ρ*) to investigate a possible relationship between interfractional organ position variation and age, height and weight, and between intrafractional right hemidiaphragm motion and age, height, and weight. As age, height and weight were highly correlated with each other (r > 0.95), we only reported on the correlations with age.

Based on the maximum age of patients treated with GA (5.4 years), we defined a subcohort of all patients < 5.5 years treated with and without GA (N_pat_=75). In this subcohort we investigated, using the Mann-Whitney U-test (Bonferroni’s corrected *p* < 0.008), if interfractional group mean position variations of each structure in patients treated with GA (N_pat_=40, age range 0.5–5.4 years) differed from patients treated without GA (N_pat_=35, age range 1.0–5.4 years). In addition, using the Mann-Whitney U-test, we tested if position variations in patients ≥ 5.5 years were significantly different compared to patients < 5.5 years treated without GA (Bonferroni’s corrected *p* < 0.008). The Mann-Whitney U-test was also used for subcohort analysis of intrafractional motion results of the right hemidiaphragm, and as only one structure was assessed, a *p* value < 0.05 was considered significant. All statistical analyses were performed using the software package RStudio [16].

## Results

### Interfractional position variation

**Fig. 2:**
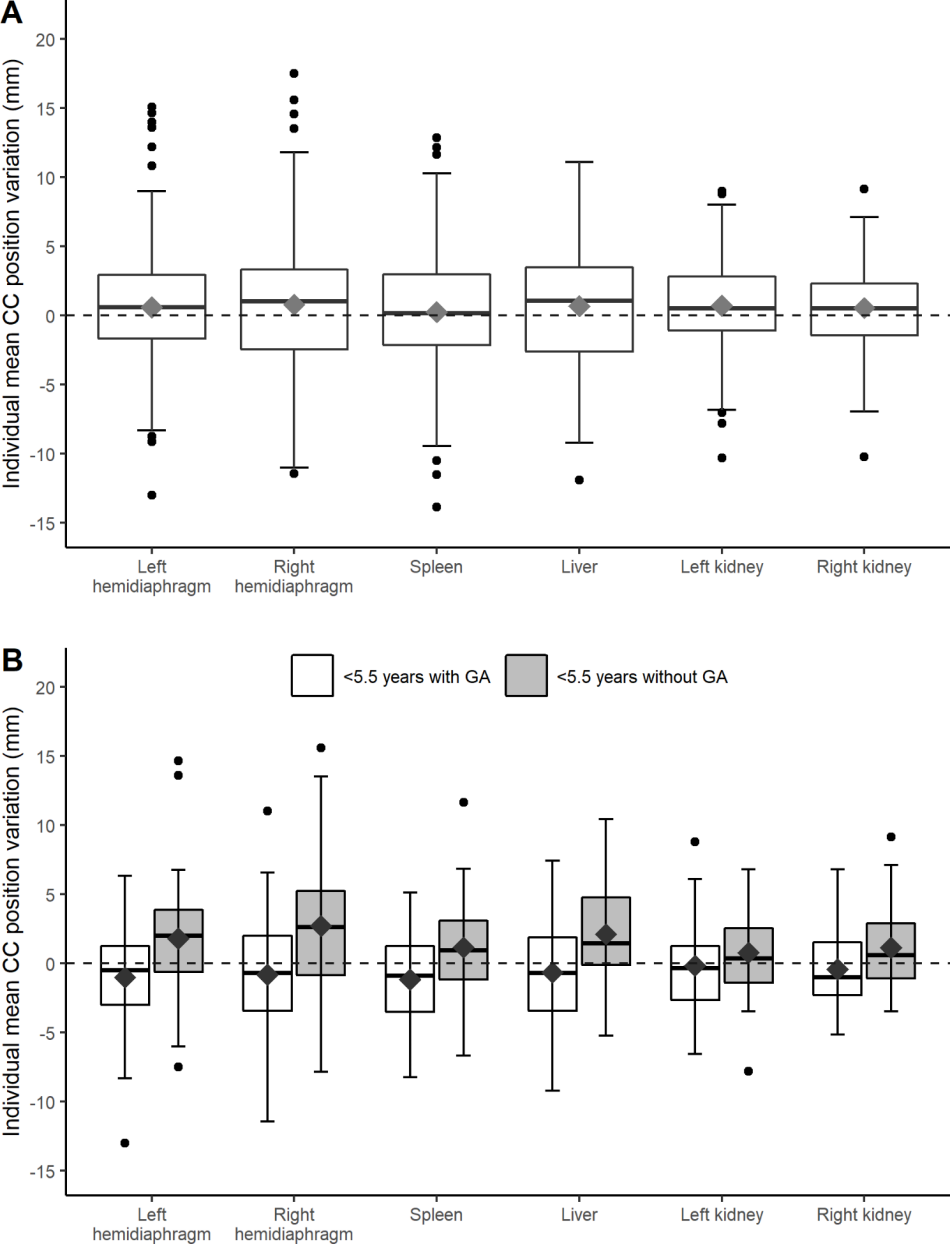
Boxplots showing the individual interfractional mean position variations relative to the refCT in CC direction for all structures, for patients treated with radiotherapy to the thoracic and/or abdominal region. Panel A shows the results of the whole cohort (N_pat_=189), and panel B of patients < 5.5 years treated with GA (white, N_pat_=40) and without GA (grey; N_pat_=35). The diamonds represent the group means. Horizontal bars, boxes, and whiskers represent medians, 50th percentiles (inter quartile range (IQR)), and the highest (lowest) value within 1.5xIQR, respectively. Solid circles denote outliers. The dotted line represents the refCT which differentiates opposite directions, where + and - signs represent caudal/right/posterior and cranial/left/anterior directions, respectively. Abbreviations: refCT, reference computed tomography scan; CC, cranio-caudal; GA, general anesthesia

Interfractional position variation of the left and right hemidiaphragm was quantified in 158/189 (83.6%) patients, and position variation of the spleen, liver, left and right kidney in 153 (81.0%), 151 (80.0%), 145 (76.7%) and 144 (76.2%) patients, respectively. After the automated two-step registration and visual inspection, 217/8707 (3.8%) registrations needed to be manually corrected. Since patient position variations were largest in CC direction, we report these results in this section; the results for LR and AP directions are presented in Additional File 1.2 (Fig. 1.2.1). Figure 2.A shows the distribution of the individual means and group mean interfractional position variation in CC direction. For all patients (N_pat_=189) and all structures, the group means of the interfractional position variations ranged between 0.2 and 0.8 mm. Ranges of individual means were widest for the left and right hemidiaphragm. The one-sample Wilcoxon signed-rank test showed that for each structures the group mean position variations were not significantly different from the reference value 0 (adjusted *p* > 0.008), meaning that there was no systematic set-up error in the position of the structures relative to the refCT. For all structures, position variations were not significantly different between patients ≥ 5.5 years and patients < 5.5 treated without GA (Mann-Whitney U test; adjusted p > 0.008).

Figure 2.B shows the distribution of individual means and the group mean interfractional position variations of the patients < 5.5 years treated with and without GA. The group mean position variations relative to the refCT were smaller in patients treated with GA compared to patients treated without GA. The Mann-Whitney U test showed that only the mean position variations of the right hemidiaphragm in patients < 5.5 years treated with GA were significantly different from patients < 5.5 years treated without GA (p = 0.004). The interfractional position variations of the left hemidiaphragm and abdominal organs were not significant different between the two subcohorts.

Table 2 presents the group mean position variations and ranges, and the systematic and random errors in CC direction. Additional File 1.3 presents the results for LR and AP directions (Table 1.3.1), and the group median for all directions (Table 1.3.2). In CC direction, the systematic and random errors for the whole cohort and the subcohorts were largest for the left and right hemidiaphragm, spleen and liver. For patients < 5.5 years treated with GA, the systematic and random errors were smaller for all structures compared to the patients < 5.5 years treated without GA. Both systematic and random errors for the spleen and liver were larger in patients ≥ 5.5 years than in patients < 5.5 years treated without GA, but no significant differences in position variations were found (Bonferroni’s adjusted p > 0.008). We found weak and non-significant (ρ < 0.20; p ≥ 0.05) correlations between interfractional position variation and age for all structures, except for the left kidney. The positon variation of the left kidney showed a borderline significant (p = 0.047), but weak (*ρ* = 0.17) correlation with age.

**Table 2:** Interfractional position variation and intrafractional motion results in cranial-caudal (CC) direction

		Age (years)											
		0.4–17.9			≥ 5.5–17.9^a^			< 5.5 with GA^a^			< 5.5 without GA^a^		
		Interfractional position variation											
	N_patients_	189			114			40			35		
	(mm)	M [range]	Σ	σ	M [range]	Σ	σ	M [range]	Σ	σ	M [range]	Σ	σ
Left hemidiaphragm		0.6 [-13.0–15.1]	4.6	3.2	0.9 [-9.1–15.1]	4.6	3.4	-1.0 [-13.0–6.3]	3.8	2.8	1.8 [-7.5–14.7]	4.8	3.4
Right hemidiaphragm		0.8 [-11.0–17.5]	5.0	3.4	0.8 [-11.0–17.5]	5.0	3.6	-0.8 [-11.4–11.0]	4.3	3.0	2.7 [-7.8–15.6]	5.4	3.3
Spleen		0.2 [-13.9–12.8]	4.6	3.4	0.6 [-13.9–12.8]	5.4	3.7	-1.2 [-8.2–5.1]	3.3	2.8	1.1 [-6.7–11.7]	3.7	3.4
Liver		0.7 [-11.9–11.1]	4.4	3.3	0.7 [-11.9–11.1]	4.5	3.5	-0.7 [-9.2–7.4]	3.8	2.9	2.1 [-5.2–10.4]	4.3	3.2
Left kidney		0.7 [-10.3–9.0]	3.4	2.5	1.0 [-10.3–9.0]	3.4	2.3	-0.2 [-6.6–8.8]	3.3	2.7	0.8 [-7.8–6.8]	3.4	3.0
Right kidney		0.5 [-10.2–9.1]	3.0	2.7	0.7 [-10.2–5.9]	2.9	2.7	-0.4 [-5.1–6.8]	2.8	2.3	1.1 [-3.5–9.1]	3.3	3.3
		Intrafractional motion											
	N_patients_	79			56			12			11		
	(mm)	M [range]	SD	σ	M [range]	SD	σ	M [range]	SD	σ	M [range]	SD	σ
Right hemidiaphragm		9.5 [3.5–16.1]	2.7	1.9	10.3 [6.9–16.1]	2.4	2.1	7.3 [3.5–10.8]	2.3	1.2	8.3 [5.6–12.3]	2.4	1.7

Note: Patients were treated for abdominal, thoracic and/or craniospinal tumors. Caudal and cranial directions are represented by + and -, respectively.

^a^Subcohorts were defined based on the maximum age of patients treated with GA (< 5.5 years).

M, group mean; range, range of individual means; Σ, systematic error; σ, random error; SD, standard deviation; GA, general anesthesia

### Intrafractional diaphragm motion

**Fig. 3:**
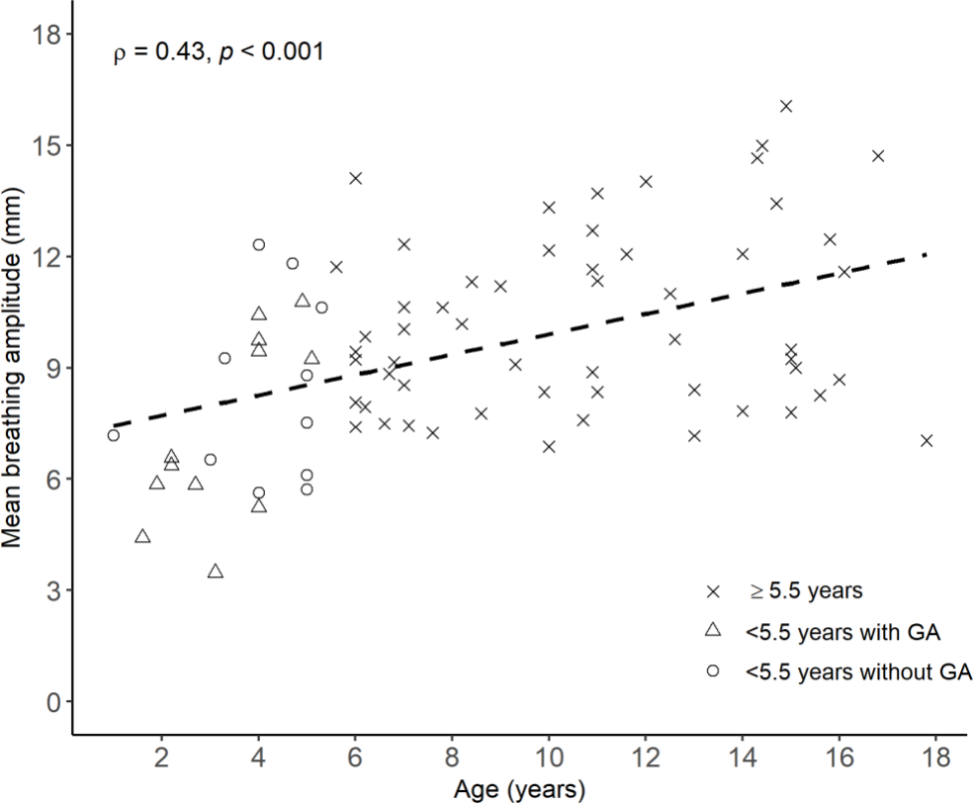
The correlation (Spearman’s ρ; significance level p < 0.05) between the individual mean breathing amplitude of the right hemidiaphragm and age. The dashed line is the regression line. The crosses represent patients ≥ 5.5 years (N_pat_=56). Triangles and circles represent patients < 5.5 years treated with GA (N_pat_=12) and without GA (N_pat_=11), respectively.

In the subgroup of 79 patients, the group mean of the breathing amplitude was largest in patients ≥ 5.5 years, and smallest in patients < 5.5 years treated with GA (Table 2). The Mann Whitney-U test showed that the difference in mean breathing amplitudes between patients ≥ 5.5 years and patients < 5.5 years without GA was significant (p = 0.02). However, the same statistical test showed that the group means of the breathing amplitudes were not significantly different between patients < 5.5 years treated with and without GA (p = 0.37). Furthermore, we found a moderate (ρ = 0.43), but significant (p < 0.001) correlation between breathing amplitude and age (Fig. 3).

## Discussion

In this retrospective multicenter study, we investigated diaphragm and abdominal motion during radiotherapy in a large cohort of 189 children. The quantification of interfractional position variations of the left and right hemidiaphragm and four abdominal organs showed that variations were largest in CC direction, with large differences between patients. For all structures, we found no correlation between interfractional position variations and age, but interfractional position variations of the right hemidiaphragm in younger patients treated with GA were significantly different compared to those of the same age treated without GA. Intrafractional motion quantification of the right hemidiaphragm in a subcohort of 79 children varied widely between patients, and showed that intrafractional motion of the right hemidiaphragm increased with age.

Data for this study was collected from six different institutes, resulting in the largest international cohort of pediatric cancer patients in which both interfractional position variations as intrafractional motion was quantified. Our recently conducted systematic review on organ motion, margin sizes and delineation variability in pediatric radiotherapy showed that seven previous studies reported on interfractional position variations [14]. These studies included 9 to 45 pediatric patients, with a total of 165 [14], which is still a considerably smaller number compared to the 189 children in this study. For a part of the AMC, UMCG and UMCN patients interfractional diaphragm and kidney position variations were previously analyzed [7], but the vast majority (79%) of this cohort consisted of newly included patients. Additionally, we succeeded to quantify position variation of the spleen and liver for the whole cohort. The AMC patients included for intrafractional motion analyses were also previously reported on [11, 13]; in this study we doubled the number of patients included for intrafractional motion analyses. Furthermore, direct comparison of previous study results was hampered due to differences in patient cohorts, quantification methods and reported results. We thus addressed the need for organ motion quantification in a large number of pediatric patients, as has been generally recommended in a number of papers [6, 7, 10, 12, 13]. Hence, our study is the first to quantify interfractional position variations of both hemidiaphragms and four abdominal organs using consistent quantification methods in the largest pediatric patient cohort.

Similar to our results, previous studies reporting on interfractional left and/or right kidney motion found absolute mean position variations ≤ 1 mm [7–9, 17]. Furthermore, we confirmed that position variations were largest in CC direction for all organs, as previously reported [7–9, 18]. This suggests the need to apply anisotropic rather than isotropic margins [7, 8]. In concordance with previous reported results [7, 9], our current analyses showed no significant correlation between organ position variations and age. We also investigated correlations between position variations and height or weight, and these variables were not correlated, similar to age. Thereby, not unexpectedly, age, height and weight were highly correlated with each other (r > 0.95; results not shown).

As reported in our systematic review [14], four smaller studies reported on intrafractional diaphragm motion in a total of 92 children (range 12 to 45 patients per study) during radiotherapy [6, 10, 11, 13]. In concordance with Pai Panandiker et al. [6], we found smaller breathing amplitudes in younger children compared to older children. Furthermore, we found that intrafractional diaphragm motion increases with age, similar to previous reported results [6, 12]. Despite these findings, direct comparison of the results is hampered as these studies used different quantification methods [6, 12].

GA can be used to immobilize young children during radiotherapy [19], but its application varies in clinical practice. In our cohort, interfractional group mean position variations in the 40 patients treated with GA differed from the 35 patients treated without GA, with significant differences for the right hemidiaphragm. This resulted in smaller systematic and random errors in patients treated with GA for all structures. Even though we only found significant differences for the right hemidiaphragm, the mean position variations for each structure in patients treated with GA tended to be in the cranial direction (negative values) in reference to the refCT, but in caudal direction (positive values) for the younger patients treated without GA and also for the whole cohort (Fig. 2). However, we did not find an explanation for this observation. The use of GA could have caused this difference in direction, and results could be comparable between the groups if this difference was not taken into account. However, patients were treated in six different institutes, and additional information on the GA protocols was not available. CT scans were also acquired with GA, however, no additional CT scans without GA or further information regarding treatment circumstances was available, and there is no evidence that the use of GA caused the difference in (direction of) mean position variations.

We found significantly smaller breathing amplitudes of the right hemidiaphragm in the 23 patients < 5.5 year compared to the 56 patients ≥ 5.5 years, which is comparable to previous reported results [6]. Furthermore, for all 79 patients, including those treated with GA, we found a significant correlation between breathing amplitude and age. Because GA alters the lung volume and pulmonary gas exchange, the use of GA could have affected the breathing amplitude. However, we found no significant differences between patients < 5.5 years treated with (N_pat_=12) and without GA (N_pat_=11), similar to results Kannan et al. found [10], and this indicates that GA had no considerable impact on the breathing amplitude.

As organ borders are more prone to organ deformations, we used the COM of the abdominal organs to quantify position variations. The effect or organ deformations on the COM coordinates is expected to be small, and therefore we did not take this into account in our analysis. Guerreiro et al. quantified position variations of the spleen, liver and one kidney (left or right after nephrectomy) also based on the COM, and found smaller ranges of mean interfractional position variations for all organs [8]. They used an average 4DCT scan with a slice thickness of 3 mm to be registered with daily CBCTs, whereas we used 3DCTs acquired following institutes’ protocols, with slice thicknesses ranging from 2.0 to 7.5 mm. Larger slice thickness could have led to larger uncertainties, and subsequently to an overestimation of standard deviations in CC direction in our analyses. Furthermore, a CBCT is acquired with a slower gantry rotation and averages the motion during image acquisition in one 3D image, whereas a 3DCT acquisition time is short and provides anatomical information at a short interval during the breathing cycle. This introduces an uncertainty regarding the reference position of the organs [20]. The use of magnetic resonance (MR) guided (adaptive) treatment systems has emerged, and MR-guided techniques offer superior resolution and soft tissue contrast, which could provide more accurate information on organ motion [12, 21]. Furthermore, the use of a nonionizing imaging technique can be promising for pediatric patients as this avoids radiation exposure. This is of particular concern in children as radiation exposure contributes to the risk of developing late treatment-related adverse effects, including second cancers [22, 23].

Since all imaging data was acquired based on institutes’ protocols, CTs and CBCTs were acquired with different parameters. Furthermore, the number of CBCTs analyzed per patient ranged from 2 to 35, meaning that single measurements weighted more in patients with less CBCTs. Therefore, we also performed the analysis when data was weighted with the number of CBCTs, but this showed no significant differences. We quantified interfractional position variations using an automated two-step registration. If an incorrect automatic registration of the organ was observed, and re-registration resulted in similar irregularities, the registration was manually corrected. In these cases (3.8%), the risk of observer bias cannot be ruled out. In case of doubt, a second observer was consulted.

Furthermore, children included in our study were treated on different body sites, with different protocols and treatment parameters. Most patients were treated in supine position, but three patients, who were included for both inter- and intrafractional analyses, were treated in prone position. Prone positioning could have caused more consistent breathing patterns [24], but because interfractional position variations and intrafractional motion results in these patients showed no significant differences with the rest of the cohort, their results were included in our analysis. We quantified intrafractional motion of the right hemidiaphragm using the AS method. Three patients were excluded from analysis because the breathing cycle could not be tracked. The observed amplitudes on the 2D AS images were too small. It seems like patients were (unintentionally) holding their breath, however, we could not verify this assumption with the available patient information.

This large multicenter cohort, availability of an extensive number of CBCTs (N_CBCT_=2051), and quantification using a consistent method, enabled a good estimation of inter- and intrafractional systematic and random errors. Our calculated systematic and random errors can be applied in the PTV or PRV margin recipes, to calculate safety margin sizes for spleen, liver and both kidneys [4, 5]. Differences in systematic and random errors between patients ≥ 5.5 years and patients < 5.5 years (whether or not treated with GA) were small, but can lead to differences in calculated margin sizes [4]. Based on the group mean inter- and intrafractional results, and the systematic and random errors, calculated margin sizes are to be expected smaller for patients < 5.5 years. However, we also showed that inter- and intrafractional results varied widely between patients, suggesting that patient-specific margin sizes should be applied. Patient-specific margin sizes can be achieved by adjusting the treatment plan based on the interfractional position variations of e.g. the first couple of fractions. Next to such offline adaptive procedure, online adaptive radiotherapy deserves a role in the treatment of pediatric patients [25].

In this study, we comprehensively analyzed interfractional position variations of both hemidiaphragms and four abdominal organs in three directions. Additionally, we quantified intrafractional motion of the right hemidiaphragm in CC direction. Previous studies have investigated to use diaphragm motion as a surrogate for abdominal organ motion, but respiratory-induced diaphragm motion is not necessarily correlated with kidney or tumor motion [7, 11, 26]. Therefore, diaphragm motion may not be a sufficient surrogate for abdominal organ motion, and if margins for abdominal organs would be calculated based on our intrafractional systematic and random errors of the right hemidiaphragm an under- or overestimation of margin sizes is to be expected. Furthermore, to properly calculate and recommend margin sizes, not only interfractional position variations and intrafractional breathing motion should be considered, but also other components such as delineation variability and set up variations.

## Conclusion

In this large cohort of 189 children, we confirmed that interfractional diaphragm and organ position variations were largest in CC direction. Interfractional variations did not correlate with age, but systematic and random errors were smaller for younger patients treated with GA. Breathing amplitudes increased with age, but there was no impact of GA on breathing amplitude. However, the large variation of interfractional position variations and breathing amplitudes between patients advocates the need for a patient-specific margin approach.

## Electronic supplementary material

Below is the link to the electronic supplementary material.

Supplementary Material 1

## Data Availability

The datasets used and/or analyzed during the current study are available from the corresponding author on reasonable request.

## Abbreviations

GTV: Gross target volume
CTV: Clinical target volume
PTV: Planning target volume
OAR: Organs at risk
PRV: Planning risk volume
(4D)CT: (Four-dimensional) Computed tomography
CBCT: Cone-beam computed tomography
MRI: Magnetic resonance imaging
GA: General anesthesia
refCT: Reference computed tomography
ROI: Region of interest
COM: Center of mass
CC: Cranial-caudal
LR: Left-right
AP: Anterior-posterior
AS: Amsterdam Shroud
SD: Standard deviation
M: Group mean
Σ: Systematic error
σ: Random error
IQR: Inter quartile range
