# Supplementary material for: Diaphragm and abdominal organ motion during radiotherapy: a comprehensive multicenter study in 189 children
Source: Radiat Oncol. 2023 Jul 13;18:119. doi: 10.1186/s13014-023-02307-3 (PMC10347831; doi:10.1186/s13014-023-02307-3)
Supplement: Supplementary file 1 — Supplementary Material 1 [file 13014_2023_2307_MOESM1_ESM.pdf]

Additional file 1.1 – Formulas

1.1.A Equations interfractional position variation analysis

The equations to calculate the group mean position variation (M_g), the group systematic (Σ) (1) and group random (σ) (2) errors for interfractional position variations in cranial-caudal (CC), left-right (LR) and anterior-posterior (AP) direction.

$$M_g = \frac{\sum_i M_i}{N_p} \qquad (1)$$

$$\Sigma = \sqrt{\frac{\sum_i (M_i - M_g)^2}{N_p - 1}} \qquad (2)$$

$$\sigma = \sqrt{\frac{1}{N_p} \sum_i SD_i^2} \qquad (3)$$

With M_i = mean position variation of patient i

N_p = number of patients

SD_i = standard deviation of the position variation of patient i

1.1.B Equations intrafractional motion analysis

The equations to calculate the group mean breathing amplitude (A_g) (6), the group standard deviation (SD_g) (8) and group random error (σ) (9) for the breathing amplitude in CC direction.

$$A_{i,f} = \frac{\sum_j (peak_{ex,i,f,j} - peak_{in,i,f,j})}{N_{b,i,f}} \qquad (4)$$

$$A_i = \frac{\sum_f A_{i,f}}{N_{i,f}} \qquad (5) \qquad \text{and} \qquad A_g = \frac{\sum_i A_i}{N_p} \qquad (6)$$

$$SD_i = \sqrt{\frac{\sum_f (A_{i,f} - A_i)^2}{N_{i,f} - 1}} \qquad (7) \qquad \text{and} \qquad SD_g = \sqrt{\frac{\sum_i (A_i - A_g)^2}{N_p - 1}} \qquad (8)$$

$$\sigma = \sqrt{\frac{1}{N_p} \sum_i SD_i^2} \qquad (9)$$

With $A_{i,f}$ = mean breathing amplitude for fraction f of patient i

$peak_{ex,i,f,j}$ = peak end-expiration for breathing cycle j of fraction f of patient i

$peak_{in,i,f,j}$ = peak end-inspiration for breathing cycle j of fraction f of patient i

$N_{b,i,f}$ = number of breathing cycles of fraction f of patient i

A_i = mean breathing amplitude over all fractions of patient i

$N_{i,f}$ = number of fractions of patient i

SD_i = standard deviation of the breathing amplitude over all fractions of patient i

Additional file 1.2 – Interfractional mean position variation

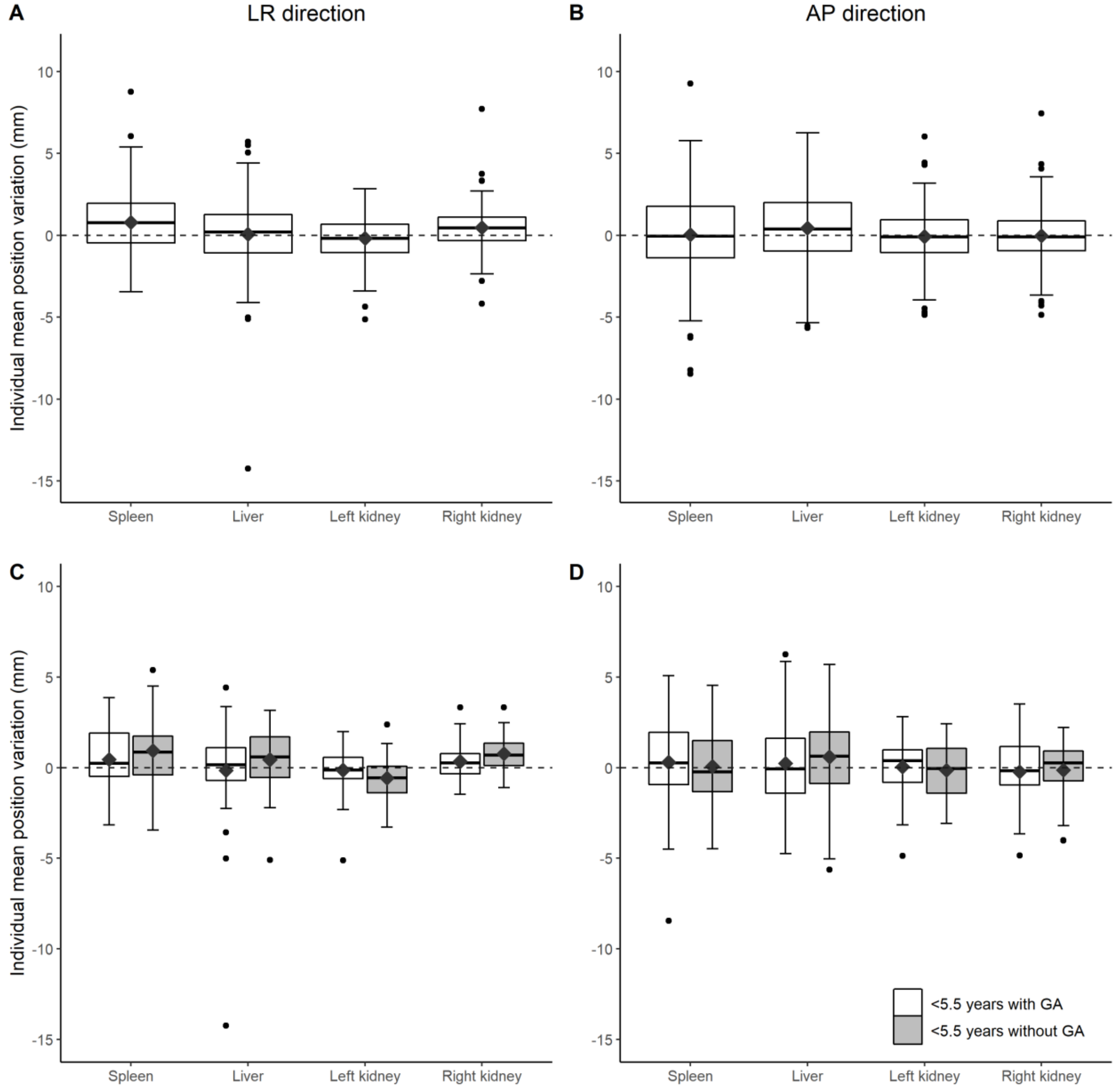

Fig. 1.2.1 Boxplots showing per structure the individual interfractional mean position variations relative to the refCT in LR (A, C) and AP (B, D) direction, for pediatric patients treated with radiotherapy to the thoracic and/or abdominal region. Panels A and B show results of the whole cohort (N_{pat}=189), and panels C and D of patients <5.5 years treated with GA (white; N_{pat}=*40*) and without GA (grey; N_{pat}=*35*). The diamonds represent the group means. Horizontal bars, boxes, and whiskers represent medians, 50th percentiles (inter quartile range (IQR)), and the highest (lowest) value within 1.5xIQR, respectively. Solid circles denote outliers. The dotted dashed line represents the refCT, which differentiates opposite directions, where + and - represent caudal/right/posterior and cranial/left/anterior directions, respectively. Abbreviations: refCT, reference computed tomography scan; LR, left-right; AP, anterior-posterior; GA, general anesthesia

Additional file 1.3 – Interfractional position variation results

Table 1.3.1 Interfractional position variation results in left-right (LR) and anterior-posterior (AP) directions.

	Age (years)											
	0.4 – 17.9			≥5.5 – 17.9[1]			<5.5 with GA[1]			<5.5 without GA[1]		
$N_{patients}$	189			114			40			35		
(mm)	M [range]	Σ	σ	M [range]	Σ	σ	M [range]	Σ	σ	M [range]	Σ	σ
LR direction												
Spleen	0.8 [-3.5–8.8]	1.9	1.8	0.9 [-3.2–8.8]	2.0	1.9	0.5 [-3.2–3.9]	1.7	1.7	0.9 [-3.5–5.4]	1.8	1.4
Liver	0.1 [-14.2–5.7]	2.3	1.8	0.0 [-4.5–5.7]	2.3	2.0	-0.2 [-14.2–4.4]	2.9	1.7	0.4 [-5.1–3.2]	1.7	1.7
Left kidney	-0.2 [-5.1–2.8]	1.4	1.1	-0.1 [-4.4–2.8]	1.4	1.1	-0.1 [-5.1–2.0]	1.3	1.2	-0.6 [-3.3–2.4]	1.3	1.2
Right kidney	0.5 [-4.2–7.7]	1.3	1.1	0.4 [-4.2–7.7]	1.5	1.1	0.3 [-1.5–3.3]	1.1	1.4	0.8 [-1.1–3.3]	1.1	0.8
AP direction												
Spleen	0.1 [-8.5–9.3]	2.7	2.4	-0.1 [-8.2–9.3]	3.0	2.6	0.3 [-8.5–5.1]	2.5	1.9	0.1 [-4.5–4.5]	2.2	2.4
Liver	0.5 [-5.6–6.3]	2.5	2.0	0.5 [-5.5–6.2]	2.5	2.2	0.2 [-4.8–6.3]	2.6	1.7	0.6 [-5.6–5.7]	2.4	1.8
Left kidney	-0.1 [-4.9–6.0]	1.7	1.7	-0.1 [-4.7–6.0]	1.8	1.6	0.0 [-4.9–2.8]	1.7	1.8	-0.1 [-3.1–2.4]	1.5	1.9
Right kidney	0.0 [-4.9–6.0]	1.8	1.7	0.1 [-4.7–6.0]	1.9	1.9	-0.2 [-4.9–2.8]	2.0	1.4	-0.1 [-3.1–2.4]	1.6	1.7

Note: Patients were treated for abdominal, thoracic and/or craniospinal tumors. Right/posterior and left/anterior directions are represented by + and -, respectively.
[1]Subcohorts were defined based on the maximum age of patients treated with GA (<5.5 years).
M, group mean; range, range of individual means; Σ, systematic error; σ, random error; GA, general anesthesia

Table 1.3.2 The median interfractional position variations in cranial-caudal (CC), left-right (LR) and anterior-posterior (AP) directions.

	Age (years)											
	0.4 – 17.9			≥5.5 – 17.9[1]			<5.5 with GA[1]			<5.5 without GA[1]		
$N_{patients}$	189			114			40			35		
Median (mm)	CC	LR	AP	CC	LR	AP	CC	LR	AP	CC	LR	AP
Left hemidiaphragm	0.6	-	-	0.9	-	-	-0.5	-	-	2.0	-	-
Right hemidiaphragm	0.7	-	-	0.8	-	-	-0.7	-	-	2.6	-	-
Spleen	0.1	0.8	0.0	0.3	0.9	-0.1	-0.9	0.2	0.3	0.9	0.8	-0.2
Liver	1.1	0.2	0.4	1.3	0.0	0.7	-0.7	0.1	-0.1	1.5	0.6	0.6
Left kidney	0.5	-0.2	-0.1	1.1	0.1	-0.3	-0.3	-0.1	0.4	0.3	-0.6	-0.1
Right kidney	0.5	0.5	-0.1	0.8	0.4	-0.3	-1.0	0.3	0.4	0.6	0.7	-0.1

Note: Patients were treated for abdominal, thoracic and/or craniospinal tumors. Caudal/right/posterior and cranial/left/anterior directions are represented by + and -, respectively.

[1]Subcohorts were defined based on the maximum age of patients treated with GA (<5.5 years).

GA, general anesthesia
